# Analysis of genotype diversity and evolution of Dengue virus serotype 2 using complete genomes

**DOI:** 10.7717/peerj.2326

**Published:** 2016-08-24

**Authors:** Vaishali P. Waman, Pandurang Kolekar, Mukund R. Ramtirthkar, Mohan M. Kale, Urmila Kulkarni-Kale

**Affiliations:** 1 Bioinformatics Centre, Savitribai Phule Pune University (formerly University of Pune), Pune, Maharashtra, India; 2 Department of Statistics, Savitribai Phule Pune University (formerly University of Pune), Pune, Maharashtra, India

**Keywords:** Dengue virus serotype 2, Bioinformatics, Comparative genomics, Evolution, Population genetics, Molecular phylogeny, Genotype/genetic diversity, Recombination, Selection pressure, Genetic structure

## Abstract

**Background:**

Dengue is one of the most common arboviral diseases prevalent worldwide and is caused by Dengue viruses (genus *Flavivirus,* family *Flaviviridae*). There are four serotypes of Dengue Virus (DENV-1 to DENV-4), each of which is further subdivided into distinct genotypes. DENV-2 is frequently associated with severe dengue infections and epidemics. DENV-2 consists of six genotypes such as Asian/American, Asian I, Asian II, Cosmopolitan, American and sylvatic. Comparative genomic study was carried out to infer population structure of DENV-2 and to analyze the role of evolutionary and spatiotemporal factors in emergence of diversifying lineages.

**Methods:**

Complete genome sequences of 990 strains of DENV-2 were analyzed using Bayesian-based population genetics and phylogenetic approaches to infer genetically distinct lineages. The role of spatiotemporal factors, genetic recombination and selection pressure in the evolution of DENV-2 is examined using the sequence-based bioinformatics approaches.

**Results:**

DENV-2 genetic structure is complex and consists of fifteen subpopulations/lineages. The Asian/American genotype is observed to be diversified into seven lineages. The Asian I, Cosmopolitan and sylvatic genotypes were found to be subdivided into two lineages, each. The populations of American and Asian II genotypes were observed to be homogeneous. Significant evidence of episodic positive selection was observed in all the genes, except NS4A. Positive selection operational on a few codons in envelope gene confers antigenic and lineage diversity in the American strains of Asian/American genotype. Selection on codons of non-structural genes was observed to impact diversification of lineages in Asian I, cosmopolitan and sylvatic genotypes. Evidence of intra/inter-genotype recombination was obtained and the uncertainty in classification of recombinant strains was resolved using the population genetics approach.

**Discussion:**

Complete genome-based analysis revealed that the worldwide population of DENV-2 strains is subdivided into fifteen lineages. The population structure of DENV-2 is spatiotemporal and is shaped by episodic positive selection and recombination. Intra-genotype diversity was observed in four genotypes (Asian/American, Asian I, cosmopolitan and sylvatic). Episodic positive selection on envelope and non-structural genes translates into antigenic diversity and appears to be responsible for emergence of strains/lineages in DENV-2 genotypes. Understanding of the genotype diversity and emerging lineages will be useful to design strategies for epidemiological surveillance and vaccine design.

## Background

Dengue infections are rising steadily with an estimate of 390 million infections worldwide every year as reported by the World Health Organization (WHO: http://www.who.int/mediacentre/factsheets/fs117/en/) and Center for Disease Control and Prevention (CDC: http://www.cdc.gov/dengue/). Severe epidemics of Dengue have been reported from more than 100 endemic countries spanning the Americas, the East Mediterranean, Western Pacific, Africa and the South-East Asia and Europe (Kyle & Harris, 2008). Dengue infections have been classified according to the levels of severity such as dengue with and without warning signs and severe dengue. Dengue infections increasingly account for heavy socio-economic burden on healthcare system in addition to the burden of 750,000 disability adjusted life years (Shepard et al., 2011; Halstead, 2007). To overcome the challenges posed by dengue infections, WHO (http://www.who.int/denguecontrol/9789241504034/en/) has set priority to reduce dengue mortality and morbidity by 50% and 25% respectively by the year 2020.

Dengue infections are caused by Dengue viruses (DENV), the members of the genus *Flavivirus* and family *Flaviviridae*. DENV are enveloped, single-stranded and positive-sense RNA viruses. The genome of DENV is of ∼11 kb long and encodes for a single open reading frame (ORF) which is flanked by 5′ and 3′ untranslated regions. The ORF encodes a single polyprotein which is cleaved into three structural (C: Capsid, M: membrane, E: envelope) and seven non-structural proteins (NS1, NS2A, NS2B, NS3, NS4A, NS4B, NS5). Currently, there are four serotypes (DENV-1 to DENV-4) of Dengue viruses based on the cross reactive assays and each serotype is known to be further divided into distinct genotypes (Chen & Vasilakis, 2011). DENV are known to undergo two types of transmission cycles called as urban and enzootic (Chen & Vasilakis, 2011). The urban cycle occurs in humans from domestic/peridomestic habitats where dengue viral transmission occurs primarily by mosquito vector species such as *Aedes albopictus* and *Aedes aegypti*. On the other hand, the enzootic cycle occurs in non-human primates of sylvatic habitats where viral transmission occurs by *Aedes taylori* and *Aedes fucifer*.

However in the absence of effective antiviral therapy, mere vector control interventions are not enough to reduce dengue transmission due to increased rural to urban migration, unplanned urbanization, population growth and the emergence of insecticide resistance in mosquitoes (Gubler DJ, 2002; WHO: http://www.who.int/denguecontrol/9789241504034/en/). Hence a broad-spectrum, efficacious, safe and cost-effective dengue vaccine needs to be developed to control the spread and prevention of dengue in endemic areas.

The diversity amongst genotypes and serotypes of DENV is one of the major challenges in the development of tetravalent vaccine. The high genetic diversity of dengue virus serotypes is mainly ascribed to its high mutation rate caused by error-prone RNA-dependent RNA polymerase, which lacks proofreading activity and generates approximately one mutation per round of genome replication (Chen & Vasilakis, 2011). Genetic recombination is also known to cause intra-serotype genetic variation in DENV (Perez-Ramirez et al., 2009; Craig et al., 2003; Uzcategui et al., 2001; Holmes & Twiddy, 2005).

Among all the four serotypes, DENV-2 is the most frequent cause of dengue epidemic worldwide and is known to be associated with severe dengue cases (Cologna, Armstrong & Rico-Hesse, 2005; Rico-Hesse, 2003). DENV-2 is divided into six genotypes such as Asian I (AI), Asian II (AII), Cosmopolitan (C), American (AM), Asian/American (AA) and sylvatic (S), based on phylogeny of envelope gene sequences (Chen & Vasilakis, 2011; Twiddy et al., 2002). In view of rapid migration and high mutation rate, the genotypes of DENV-2 are constantly undergoing evolutionary changes and there is possibility of emergence of new lineages (Chen & Vasilakis, 2011). Complete genome sequence data is available for DENV-2 strains across the world. The present study aims to understand the diversity of known DENV-2 genotypes and to detect the emerging genotypic lineages of DENV-2. Complete genomes of DENV-2 isolates were analyzed using both the phylogenetic and a Bayesian-based population genetics approach, implemented in the STRUCTURE program (Pritchard, Stephens & Donnelly, 2000; Falush, Stephens & Pritchard, 2003). The STRUCTURE program accounts for recombination (Falush, Stephens & Pritchard, 2003) and is commonly used for inference of genetic structure in viruses (Szmaragd & Balloux, 2007; Waman et al., 2014), bacteria (Falush et al., 2003) and higher organisms (Rosenberg et al., 2002). In the present study, the program is used to infer the DENV-2 population structure and to identify the extent of recombination (or admixture). The role of recombination, selection pressure and spatiotemporal factors in genotype diversity of DENV-2 serotype is analyzed in this study. Understanding of the genotype diversity of DENV-2 will be useful in devising efficient strategy for epidemiological surveillance, transmission control and vaccine design.

## Methods

### Dataset

The dataset consisting of 990 complete genome sequences of strains of Dengue virus serotype 2 (DENV-2) was compiled from the Dengue virus variation resource at National Center for Biotechnology Information (Resch et al., 2009). The DENV-2 serotype is divided into six known genotypes such as Asian I (AI), Asian II (AII), American (AM), Asian/American (AA), Cosmopolitan (C) and Sylvatic (S).

The genotype information was not available for the sequence entries in dataset at the Dengue virus variation resource. Therefore, genotyping of all the 990 strains of DENV-2 strains was carried out using Dengue SubTyper tool (Kolekar, Kale & Kulkarni-Kale, 2012). The GenBank accession numbers (as of November, 2015), spatiotemporal distribution and genotype information of the entries in the data set are provided in File S1.

### Inference of genetic structure of DENV-2 population

Genetically distinct subpopulations (or lineages) within DENV-2 population were identified using Bayesian-based population genetics and phylogenetic approaches.

The steps involved in the study of genetic structure of DENV-2 population using population genetics approach include (i) multiple sequence alignment (MSA) of 990 DENV-2 genomes using MUSCLE program (Edgar, 2004) available in MEGA v6 software (Tamura et al., 2013) (ii) extraction of parsimony-informative (PI) sites from MSA using MEGA, for subsequent analyses, (iii) analysis of linkage equilibrium using LIAN v3.5 (Haubold & Hudson, 2000) and DNASP v5 programs (Librado & Rozas, 2009) (iv) identification of genetically distinct subpopulations and study the extent of admixture using a Bayesian-based clustering program called STRUCTURE (Pritchard, Stephens & Donnelly, 2000; Falush, Stephens & Pritchard, 2003) and (v) validation of genetic structure using Analysis of Molecular Variance (AMOVA) test with 1,000 permutations, the default setting in ARLEQUIN v3.11 software (Excoffier, Laval & Schneider, 2005). It should be mentioned that the geographical information was not incorporated for the clustering analysis. This protocol has been previously described and successfully used to infer genetic structure in *Rhinoviruses* (Waman et al., 2014) and DENV-4 (Waman et al., 2016). The details of each of these steps and parameters used for analysis of DENV-2 population are provided in File S2.

### Molecular phylogenetic approach

The genomic data of 990 DENV-2 strains and an out-group that includes three genome sequences of Japanese Encephalitis virus (JEV) (GenBank: NC_001437.1), West Nile virus (WNV) (GenBank: NC_001563.2) and Murray Valley encephalitis virus (MVEV) (GenBank: NC_000943.1) was compiled (Benson et al., 2013). The multiple sequence alignment of all these sequences (File S3) was used to generate phylogenetic trees, using three methods namely Neighbor-joining (NJ), Maximum likelihood (ML) and Maximum parsimony (MP) that are available in MEGAv6 (Tamura et al., 2013). Bootstrap analysis was carried out by sampling 1,000 replicates. For the visualization of phylogenetic trees, Rambaut (2009) (http://tree.bio.ed.ac.uk/software/figtree/) software was used.

### Recombination analysis

MSA of 990 DENV-2 genomes was used as input for RDP4 package (Martin et al., 2015) to identify potential recombinant sequences and their parents (major and minor) using seven recombination detection methods. These methods include RDP (Martin et al., 2015), GENCONV (Padidam, Sawyer & Fauquet, 1999), BOOTSCAN (Martin et al., 2005), MAXCHI (Smith, 1992), CHIMAERA (Posada & Crandall, 2001), SiScan (Gibbs, Armstrong & Gibbs, 2000) and 3SEQ (Boni, Posada & Feldman, 2007). A sequence is considered as a potential recombinant only if it is detected as significant (with *p-value* cutoff of 0.00001) by at least two methods stated above. The multiple comparison correction setting option was used.

### Selection pressure analysis

Potential recombinants (identified using RDP4 package) were excluded from selection pressure analysis and thus the dataset of 964 DENV-2 genomes was used. Using these entries, eleven separate datasets corresponding to each of the 10 individual coding genes (C, M, E, NS1, NS2A, NS2B, NS3, NS4A, NS4B, NS5) as well as ORF coding for the polyprotein were generated.

For each of these 11 datasets, MSA was carried out using the MUSCLE program. As the accuracy of positive selection analysis depends primarily on the quality of MSA (Privman, Penn & Pupko, 2012), the quality of the MUSCLE-generated codon alignments was evaluated using GUIDANCE server (Penn et al., 2010) and was observed to be best (as indicated by the GUIDANCE score of ∼1).

All the 11 datasets were independently subjected to selection pressure analysis using ML-based methods (with default *p* = 0.1) available on the Datamonkey server of HYPHY package (Pond & Frost, 2005a; Pond, Frost & Muse, 2005) These methods were Fixed Effect Likelihoods (FEL) (Pond & Frost, 2005b), Single Likelihood Ancestor Counting (SLAC) (Pond & Frost, 2005b) and Internal Fixed Effect Likelihoods (IFEL) (Pond et al., 2006). The automated model selection tool at Datamonkey server was used for selection of appropriate nucleotide substitution bias model for every dataset. The SLAC, FEL and IFEL methods detect the sites that are under pervasive positive selection across all the lineages in a phylogenetic tree. In order to detect the evidence of episodic positive selection, which affect only a small subset(s) of lineages even when the majority of the lineages are under purifying selection, Mixed Effects Model of Evolution (MEME) method was used (Murrell et al., 2012).

### Genotype-phenotype correlation

Amino acid residues corresponding to the codons under positive selection were mapped on the DENV proteins for which three-dimensional structures are available in Protein Data Bank (PDB) (Berman et al., 2000). The structures were visualized using SwissPDB viewer 3.7 (Guex & Peitsch, 1997). The functional implications of these residues with respect to antigenicity were studied using the dataset of experimentally validated (B-cell and T-cell) epitopes archived in Immune epitope database (Vita et al., 2014).

## Results

The genetic diversity within the genotypes of DENV-2 population was analyzed using complete genome dataset of 990 strains. The dataset comprises of 273 genomic entries of Asian I (AI) genotype, 26 entries of Asian II (AII), 552 entries of Asian/American (AA), 84 entries of cosmopolitan (C), 39 entries of American (AM) and 16 entries of sylvatic (S) genotype. The spatiotemporal data was used to understand the population structure and evolution.

### Identification of genetically distinct subpopulations in DENV-2

The complete genome alignment of 990 strains of DENV-2 helped to identify 4,470 parsimony-informative sites, which were used for cluster analysis using admixture model in STRUCTURE program. The plot of *K* (number of clusters) vs Δ*K* (the rate of change of posterior probability given *K*) provided a major peak of Δ*K* at *K* = 2 (*F*_ST_ = 0.50, *p* = 0) a minor peak at *K* = 15 (*F*_ST_ = 0.82, *p* = 0) (File S4). Analysis of clustering obtained at *K* = 2 revealed that the DENV-2 population is subdivided into two main clusters based on geographic origin of strains. All the American strains of Asian/American (AA) genotype formed a distinct cluster whereas all the strains of Asian origin from other genotypes such as Asian I (AI), Asian II (AII), Cosmopolitan (C), American (AM) and Sylvatic (S) were observed to form a second cluster. The Asian strains of AA genotype were observed to be admixed having membership for both of these clusters. The genetically distinct nature of Asian and American strains of DENV-2 observed using whole genome data substantiates earlier reports based on envelope gene and genomic data (Chen & Vasilakis, 2011; Lara-Ramírez et al., 2014).

**Figure 1:**
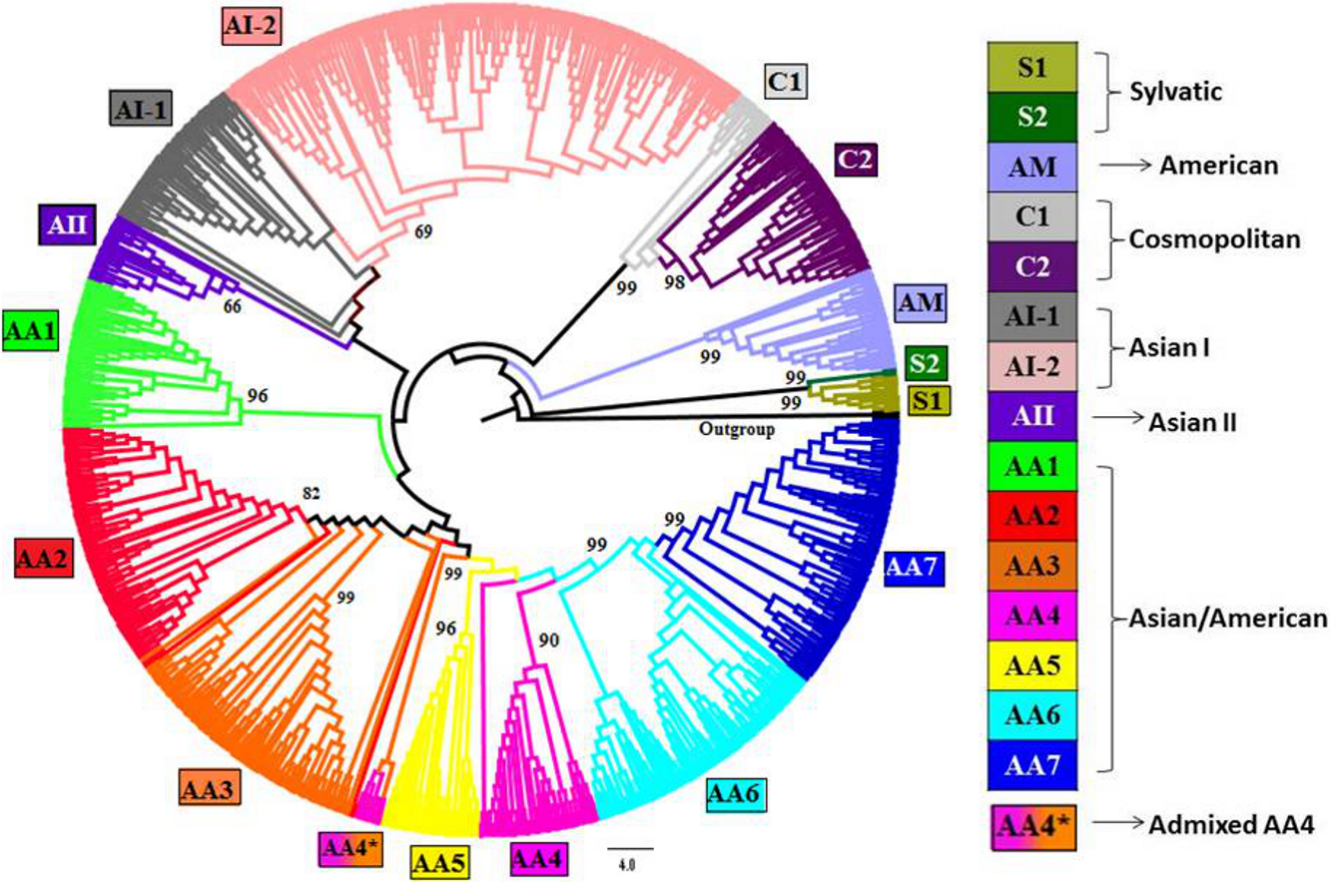
Phylogenetic tree of DENV-2 strains obtained using Neighbor-joining (NJ) method in MEGA. Complete genomes of 990 strains of DENV-2 with 1,000 bootstrap replicates were used to reconstruct phylogenetic tree using NJ method. The fifteen lineages, which are also obtained using STRUCTURE program, are depicted in the tree using color codes as indicated. The bootstrap value (%) associated with each lineage is indicated. There are two lineages (S1 and S2) of Sylvatic genotype, two lineages (C1 and C2) of the cosmopolitan genotype, two lineages (AI-1 and AI-2) of Asian I genotype and a total of seven lineages (AA1–AA7) of Asian/American genotype. The American (AM) and Asian II (AII) genotypes formed independent clusters. AA4* indicates the clade of admixed strains that were found to belong to the AA4 lineage by the STRUCTURE program.

The presence of second peak (at *K* = 15) implies that the DENV-2 population has a complex genetic structure and comprise of a total of 15 genetically distinct ‘subpopulations’ or ‘lineages’. Existence of such genetic structure is supported by the highly significant *F*_ST_ value (0.82, *p* = 0) obtained by the AMOVA test and was observed to be attributed to the subdivision of DENV-2 genotypes such as AA (7 lineages), C (2 lineages), AI (2 lineages) and S (2 lineages) whereas no subdivisions were observed in case of AM and AII genotypes. The assigned lineage for every strain in the dataset is provided in File S1.

In order to confirm the presence of such genetic structure, genome-based phylogenetic trees were also generated using NJ (Fig. 1), ML (File S5: Fig. S1) and MP (File S6: Fig. S2) methods. The tree topologies obtained using all the three methods supported presence of the 15 clusters (lineages) as identified by the population genetics approach. The subdivision of AA, AI, C and AA genotypes into respective lineages was also found to be consistent with the clustering results obtained by the STRUCTURE program. However, in case of recombinant strains, the STRUCTURE program helped to provide accurate cluster assignments which are discussed in the section describing ‘Evidence of recombination’.

In order to further analyze the heterogeneous nature and evidence of substructure in AA, C, AI and S genotypes, sublevel clustering analysis was independently carried out for each of these four genotypes. Sublevel clustering results also supported the presence of substructure and diversifying lineages within these genotypes, which are explained below.

### Genetic structure of Asian/American (AA) genotype of DENV-2

Sublevel cluster analysis of AA genotype population (of 552 strains) supported presence of two peaks i.e., a first peak at *K* = 4 (*F*_ST_ = 0.53, *p* = 0) followed by a second peak at *K* = 7 (*F*_ST_ = 0.62, *p* = 0) (File S7). Thus, the population of Asian/American genotype is subdivided into four major clusters and seven minor subpopulations/lineages. The four major clusters correspond to the strains isolated from Asia, Central America, South America and North America. The population stratification analysis of AA genotype at *K* = 7, revealed further subdivision of South and Central American clusters into additional lineages whereas Asian (AA1) and North American (AA2) subpopulations remained homogeneous and formed independent lineages. The cluster of South American strains was observed to undergo spatiotemporal diversification into three distinct lineages (AA3–AA5). AA3 lineage was found to comprise of older strains from USA (1986–1996), Brazil (1990–2000), Puerto Rico (1994–1995) and Venezuela (1991–1998). The American strains isolated during 1996–2008 from USA (1998–1999), Brazil (2000–2006), Venezuela (1996–2007), and Colombia (1999–2007) formed a distinct lineage referred to as AA4. Similarly, the AA5 lineage is characterized by modern strains from USA (1998–2007), Brazil (2007–2008), Dominican Republic (2001–2003) and Jamaica (2007). The spatiotemporal information of strains belonging to every lineage is provided in File S1.

**Figure 2:**
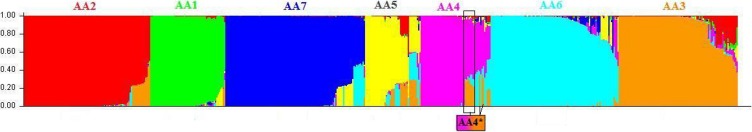
Genetic structure of Asian/American genotype obtained using the STRUCTURE program using admixture model. Sublevel cluster analysis of the dataset of 552 strains of Asian/American genotype was carried out using STRUCTURE program. The presence of seven distinct lineages are depicted as a bar plot. The seven lineages are AA1 (green), AA2 (red), AA3 (orange), AA4 (pink), AA5 (yellow), AA6 (cyan) and AA7 (blue). These lineages correspond to the clusters obtained using the NJ-based tree (Fig. 1) and have been colour-coded. The STRUCTURE program resolved the cluster assignments of admixed strains of all the seven lineages in general and that of AA4 lineage, in particular. The admixed strain of AA4 cluster, denoted as AA4*, are found to group with the strains of AA3 lineage in the NJ tree, have >0.50 membership for AA4 (indicated in pink) subpopulation and ∼0.35 membership for AA3 (orange) subpopulation.

The cluster of Central American strains was subdivided into two lineages such as AA6 and AA7. The seven lineages of AA are shown in the bar plot which is obtained using the STRUCTURE program (Fig. 2) and in the phylogenetic tree (Fig. 1). The details of strains belonging to AA1–AA7 are given in File S1. It should be noted that the STRUCTURE program assigns a membership score of 1 if an individual belongs to one particular subpopulation. An admixed (recombinant) strain is assigned with multiple membership scores (summing to 1) to indicate its membership to multiple subpopulations. The population genetics approach helped to resolve the cluster assignments of several strains from USA isolated in 1998 (GenBank: EU482545, EU482734, EU482735) and from Brazil isolated during 2000–2006 (GenBank: FJ850074, FJ850076, FJ850078, FJ850082, FJ850085, FJ850088, GQ868640, JN819419). All these strains are found to be admixed having >0.50 membership for AA4 subpopulation while ∼0.35 membership for AA3 subpopulation. Therefore, the population genetic analyses indicate that these strains belong to AA4 subpopulation (Fig. 2). In the phylogenetic tree, all these strains were observed to form an independent clade and designated as AA4*, to represent their admixture for AA3 and AA4 lineages.

### Genetic structure of Asian I (AI) genotype of DENV-2

Complete genomes of 273 strains of Asian I (AI) genotype were analyzed to infer its genetic structure. A clear peak of Δ*K* was obtained at *K* = 2 (File S8). It supports time-dependent subdivision of AI genotype into two lineages. One of the lineages was found to comprise of all the Thailand strains and only one strain from China isolated during 1985–2001 and is referred to as AI-1. The second lineage comprised of the Asian I strains isolated during 2001–2008 from countries such as Thailand, Cambodia and Vietnam, and is referred as AI-2. Two Thailand strains isolated in 1995 (GenBank: GQ868543) and 1996 (GenBank: FJ906957) also showed the membership to belong to AI-2, thus indicating their role in spread of DENV-2 in Thailand as well as other countries, where virus spread is characterized.

### Genetic structure of Cosmopolitan (C) genotype of DENV-2

Analysis using the dataset of whole *DENV-2* population helped to reveal the time-dependent subdivision of the Cosmopolitan (C) genotype into two distinct lineages (at *K* = 15). One of the cosmopolitan lineages was formed by the Indonesian strains isolated during 1975–1976 and hence referred to as older C1 lineage. The C1 lineage, however, was also found to include strains from India, Shri Lanka and Burkina Faso, isolated during 1983–2006. The other cosmopolitan lineage was formed by the Indonesian strains isolated in 1976 and 1998 as well as strains from Asian and American countries, isolated during 1998–2012 and is referred to as modern C2 lineage. Thus, the Indonesian strains of older C1 lineage seem to have played important role in spread of DENV-2 to the various Asian and American countries.

Sublevel clustering analysis using complete genomes of 84 strains of cosmopolitan genotype (File S9) also supported the heterogeneous nature of cosmopolitan genotype and revealed that the modern C2 lineage is further divided into three sub-lineages, based on spatiotemporal distribution. The strains isolated from Taiwan (during 2001–2002) and Singapore (2005–2008) formed two independent sub-clusters while the third sub-cluster comprises of cosmopolitan strains from various countries such as Australia, Guam (2001), Singapore (2004–2006 and 2009), Vietnam (2006), Brunei (2005) and China (2003, 2010).

### Genetic structure of sylvatic (S) genotype of DENV-2

Sublevel cluster analysis of the dataset (total 16) of sylvatic strains supported a clear peak of Δ*K* at *K* = 2 (File S10), which confirms the presence of two lineages within sylvatic genotype.

All the sylvatic strains isolated from African countries (such as Senegal, Burkina Faso, Cote d’Ivoire and Nigeria) were observed to form a single cluster, referred to as S1 lineage. On the other hand, only two isolates from Malaysia (GenBank: EF105379, FJ467493) were observed to form a genetically distinct lineage, referred to as S2 lineage. An earlier study also substantiates the genetically distinct nature of the sylvatic strains from Malaysia and Africa (Vasilakis et al., 2008). Apart from the difference in the geographic origin, the strains from S1 and S2 lineages were observed to have distinct hosts. The strains of S1 lineage were isolated from either the mosquito or human host whereas the S2 isolate P8-1407 (GenBank: EF105379) was isolated from a monkey-like host (Simiiformes). Thus, genetic diversity within sylvatic genotype could be attributed to both the geographic origin and to the host environment.

It is to be noted that, the sublevel clustering analysis helped to resolve the genetic differences between the two geographically distinct lineages of the sylvatic genotype, despite fewer genomic entries for sylvatic strains in DENV-2 dataset.

### Evidence of recombination

A total of 26 recombinant strains were identified (with *p* < 0.00001) using RDP4 package (Table 1). These recombinants belong to genotypes AA, AI, C and AM.

**Table 1:** Dengue virus serotype 2 (DENV-2) recombinant strains obtained using RDP4 package. A total of 26 recombinant strains were identified using RDP4 with *p*-value < 0.00001. The breakpoint positions are reported according to the position in the respective recombinant sequence. The recombination is detected using at least two of the six recombination detection methods in RDP4 program namely RDP, GENCONV, BOOTSCAN, MAXCHI, CHIMAERA, SISCAN and 3SEQ and are represented as R, G, B, M, C, S and Q, respectively. The method showing comparatively lowest *p*-value is shown and is represented as bold and the corresponding *p*-value is also reported.

Serial number	Recombinant DENV-2 strain [Genotype_ GenBank-Accession]	Major Parent [Genotype_ GenBank-Accession]	Minor Parent [Genotype_ GenBank-Accession]	Breakpoint start	Breakpoint end	Methods: RGBMCSQ	Lowest *p*-value
1	AM_GQ398257	AM_GQ868600	AA_GQ398269	4,256	6,361	RGBMCS**Q**	3.70E−140
2	AM_JQ922552	AM_JQ922550	AM_JQ922549	6,081	6,535	**R**GBMCS	1.05E−20
3	AM_JQ922553	AM_JQ922552	C_JQ922551	7,448	8,410	**R**GBMCSQ	1.97E−36
4	AM_JQ922550	AM_JQ922552	C_JQ922551	7,448	8,410	**R**GBMCSQ	1.97E−36
5	AM_AF100467	AM_EU056811	AM_GQ868588	6,000	7,947	RGB**Q**	1.23E−18
6	C_EU179859	C_EU482640	Unknown	2,985	3,975	RGBMCS**Q**	3.56E−84
7	C_FJ196854	C_JN851128	A1_FJ196851	0^*^	773	RGB**S**	1.89E−28
		C_GQ398264	A1_FJ196851	10,200	10,498^*^	RG**B**	1.55E−14
8	C_ JQ922551	C_GQ398261	AM_JQ922552	1,544	1,824	RG**B**	5.428E−08
9	AA_GQ398269	AA_GQ398300	AAF204178	4,289	6,204	RG**B**MCS	4.51E−53
10	AA_JN819408	AA_GQ868553	Unknown	10,411	10,620^*^	**R**GBMC	1.41E−69
11	AA_EU482726	AA_EU596491	AA_EU482719	5,260	8,059	G**B**MCS	1.22E−38
12	AA_JF357906	AA_EU621672	Unknown	10,466	10,783^*^	R**G**BMCQ	1.60E−113
13	AA_FJ744708	AA_JF357906	Unknown	7,502	7,567	R**B**	2.97E−08
14	AA_JN819418	AA_M20558	Unknown	10,266	10,576^*^	**R**GBMC	8.77E−78
15	AA_AF100466	AA_GQ868540	A1_AF100463	0^*^	606	**R**GBSQ	6.77E−40
16	AI_FJ196851	AI_GQ868545	C_FJ196852	4,984	5,266	RG**B**	1.82E−22
		AI_GQ868545	C_FJ196853	8,294	9,438	RG**B**MCSQ	3.33E−44
17	AI_FJ687444	AI_FJ744721	Unknown	10,277	10,363^*^	RG**B**	6.92E−27
18	AI_EU482784	AI_EU482775	C_EU482640	2,726	3,751	**R**GBMCSQ	8.50E−73
		AI_FM210211	Unknown	9,970	10,679^*^	**R**GBMC	1.59E−19
19	AI_FJ410193	AI_FM210211	Unknown	0^*^	59	R**G**B	2.79E−08
20	AI_FM210211	AI_FJ687444	AA_EU482783	9,988	10,691^*^	RG**B**MCS	3.04E−29
21	AI_GU131924	AI_FM210211	Unknown	9,955	10,557^*^	**R**GBMC	1.59E−19
22	AI_JN368476	AI_GU131930	AI_GU131897	8,188	9,896	RGBS	1.14E−12
23	AI_FJ639717	Unknown	AI_GU131897	8,317	9,306^*^	**R**GBSQ	1.14E−12
24	AI_JF730047	Unknown	AI_GU131897	8,329	9,318^*^	**R**GBSQ	1.14E−12
25	AI_GU131930	Unknown	AI_GU131897	8,317	9,306^*^	**R**GBSQ	1.14E−12
26	AI_JF730048	Unknown	AI_GU131897	8,328	9,317	**R**GBSQ	1.14E−12

**Notes.**

* Indicates that the actual breakpoint position is undetermined in subsequent recombination event.

The recombination analysis was found to be useful to resolve the uncertainty in classification of the recombinant strains (Rico-Hesse, 2003). For example, clustering of a recombinant strain namely DF907 (GenBank: FM210211) into AI-1 or AI-2 lineage was unclear in the NJ-based genome tree (bootstrap value 49%). The STRUCTURE program resolved the admixed nature of this strain and assigned it to AI-1 subpopulation as it had a major membership (of 0.90) for AI-1 and minor membership (of ∼0.10) for AA1. Further analysis using RDP4 package also supported that this strain has a major and minor parent from AI-1 and AA1 subpopulations, respectively (Table 1).

Similarly, in case of a strain belonging to AA genotype namely DENV-2/PR/50DN/1994 (GenBank: GQ398269), NJ-tree showed its clustering with strains belonging to AA2 lineage. Analysis using STRUCTURE and RDP4 (Table 1) revealed its recombinant nature, having its major parent from AA3 and minor parent from AII subpopulation (Table 1).

### Evidence of positive selection

The significant evidence (*p* < 0.05) of episodic positive selection was obtained for all the coding genes, except NS4A (Table 2).

**Table 2:** Codons of DENV-2 under episodic diversifying selection identified using MEME method. The codons under significant evidence of episodic positive selection (*p* < 0.05) were obtained in all the genes except for NS4A. The table provides MEME results such as distribution of synonymous (*α*) and non-synonymous (*β*) substitution rates over sites. The maximum likelihood estimate (MLE) of the non-synonymous rate for the branch class with *β* ≤ *α* is represented as ‘*β* −’. ‘Pr[*β* = *β* −]’ represents the MLE of the proportion of sites evolving at ‘*β* −’. The MLE of the unconstrained *β* non-synonymous rate is given as ‘*β* +’. The MLE of the proportion of sites evolving at *β* + is represented as ‘Pr[*β* = *β* +]’. The false discovery rate is controlled by the ‘*q*-value’, under the strict neutral null.

Sr. no.	Gene	Codon	*α*	*β* −	Pr[*β* = *β* −]	*β*+	Pr[*β* = *β* +]	*p*-value	*q*-value
1	C	11	0.164376	0	0.953173	21.9755	0.046827	0.005121	0.583832
2	M	147	0.159745	0	0.997987	3769.49	0.002013	5.37E−05	0.008919
3	E	91	1.7408	0.032332	0.992346	706.968	0.007654	0.009902	1
4	E	128	0	0	0.994362	305.675	0.005638	0.013207	1
5	E	192	1.61287	0	0.994973	544.37	0.005027	0.001674	0.828819
6	E	271	0.62851	0	0.998508	3578.71	0.001492	0.01428	1
7	E	340	0	0	0.998292	238.733	0.001708	0.004202	1
8	E	363	1.39999	0	0.99606	156.326	0.00394	0.00753	1
9	E	402	0.557236	0	0.996846	1009.79	0.003154	0.0285	1
10	E	455	0	0	0.996943	910.053	0.003057	0.021326	1
11	NS1	47	0.849852	0	0.996678	1630.99	0.003322	0.013512	1
12	NS1	146	0.182516	0	0.987198	92.3804	0.012802	0.015277	1
13	NS1	164	0.74287	0	0.981603	391.58	0.018397	0.000484	0.085203
14	NS1	181	0.498353	0	0.993067	176.231	0.006933	0.049911	1
15	NS1	207	0	0	0.995688	174.175	0.004312	0.000447	0.157292
16	NS1	230	1.78007	0	0.997897	182.989	0.002103	0.007617	0.893734
17	NS2A	16	0.409451	0	0.997886	2812.78	0.002114	5.39E−07	0.000118
18	NS2A	72	0.290106	0	0.993819	67.2445	0.006181	0.009687	0.351954
19	NS2A	111	0.477414	0	0.995018	32.6139	0.004982	0.019069	0.593865
20	NS2A	114	1.77037	0	0.996181	570.44	0.003819	0.005226	0.227852
21	NS2A	119	1.29922	0	0.99421	1136.87	0.00579	0.004792	0.261146
22	NS2A	140	0	0	0.998138	1625.9	0.001862	0.044979	1
23	NS2A	181	1.79665	0.205045	0.987651	757.326	0.012349	0.000884	0.09635
24	NS2A	199	0.884091	0	0.997546	168.309	0.002454	0.004221	0.306752
25	NS2B	63	1.48592	0.12202	0.992686	320.784	0.007314	0.001293	0.168124
26	NS3	14	0	0	0.86109	10.8058	0.13891	0.000166	0.102849
27	NS3	49	0	0	0.99844	216.962	0.00156	0.000191	0.059005
28	NS3	138	0.155937	0	0.998495	866.279	0.001505	0.004107	0.846078
29	NS4B	122	1.01576	0	0.995707	318.631	0.004293	0.000418	0.051883
30	NS4B	156	0.103265	0.103265	0.996932	3312.6	0.003068	0.000167	0.041538
31	NS5	5	0.546428	0.3356	0.986448	37.3205	0.013552	0.045428	1
32	NS5	6	0.151621	0	0.998617	1120.9	0.001383	0.00018	0.054024
33	NS5	25	0.934525	0	0.998355	329.819	0.001645	0.004794	0.862932
34	NS5	130	1.84523	0	0.99369	134.257	0.00631	0.031375	1
35	NS5	209	0.434112	0	0.998651	3523.06	0.001349	3.28E−07	0.000148
36	NS5	271	0	0	1.00E−09	1.65517	1	0.014739	1
37	NS5	400	0.508322	0	0.959072	11.2506	0.040928	0.022174	1
38	NS5	414	0.639643	0	0.996067	3523.04	0.003933	0.003799	0.854778
39	NS5	558	0	0	0.823609	3.9341	0.176391	0.010112	1
40	NS5	679	0	0	0.917709	2.65504	0.082291	0.023981	1
41	NS5	803	0	0	0.997137	1066.86	0.002863	1.75E−07	0.000158

Selection on four codons within NS5 (codon-5, -271, -558 and -679) was observed. In case of sylvatic genotype, episodic positive selection on two codons (codon-271 and -679) in NS5 gene appeared to be stronger on the branch leading to the members of S2 lineage (GenBank: EF105379, FJ467493), which possess Ile-271-Val and Thr-679-Val mutations. The amino acid at position 271 is part of a loop forming linker region (residues 262–272), which is known to play a regulatory role in viral replication (Zhao et al., 2015; Egloff et al., 2002). The residue at the 679^th^ position occurs within the palm domain of the NS5 protein structure [PDB: 1L9K] (Zhao et al., 2015; Egloff et al., 2002; Yap et al., 2007).

The Thr-400-Cys mutation was observed in all the members of sylvatic genotype. Interestingly, all the American strains of AA genotype possess Lysine at the 558^th^ position of NS5, whereas the Asian AA lineage (AA1) and AM strains have Lys-558-Ala and Lys-558-Glu mutations, respectively. Selection on codon-5 of NS5 was stronger on the branch leading to the cosmopolitan genotype, which carry Ile-5-Thr mutation.

Apart from NS5 gene, episodic selection on codons of non-structural genes such NS1 (codon-146), NS2A (codon-72), NS2B (codon-63) and NS4B (codon-156) was found to play an important role in the evolution of DENV-2 strains. The NS1 codon-146 is known to be part of the B-cell epitope (Tai et al., 2005) whereas the NS2A (codon-72), NS2B (codon-63) encodes for amino acids that are part of known T-cell epitopes (Malavige et al., 2012). The Asp-63-Glu mutation in NS2B was found in all the strains of the modern C2 lineage of cosmopolitan genotype. Similarly, the mutation at codon-156 of NS4B gene was observed to distinguish all the urban Asian strains (Glu-156) than that of American strains (Glu-156-Asp) of DENV-2. Thus, the transition from sylvatic to human transmission was mediated through the adaptive evolution of the non-structural genes (Twiddy, Woelk & Holmes, 2002).

Selection on two codons (codon-91 and -340) encoding for amino acids that are part of the T-cell epitope (Roehrig et al., 1994) within envelope gene appears to correlate with the spatiotemporal structure of AA genotype. The strains belonging to the three AA lineages such as AA1 (Asian), AA2 (North American), AA3 (older South American) were observed to carry Val91 and Thr340. On the other hand, the strains of modern lineages from South America (AA4, AA5) and Central America (AA6, AA7) contain Val-91-Ile and Thr-340-Met mutations.

Limited evidence of pervasive positive selection was identified when analyzed using FEL, IFEL and SLAC. It was found to be significant in case of only one codon in NS1 gene (codon-172, *p* < 0.1). The Arg-172-Lys mutation was observed in the strains belonging to AI and C genotypes.

## Discussion

DENV-2 is the prevalent serotype in worldwide dengue epidemics. The emergence of new lineages of DENV-2 genotypes has been reported earlier (Hang et al., 2010; Ernst et al., 2015; Drumond et al., 2013; Foster et al., 2004; Williams et al., 2014). The fine-scale population genomic analysis of DENV-2 carried out in this study helped not only to resolve the genotypic lineages within DENV-2 but also helped to elucidate the contribution of evolutionary (recombination, selection pressure) and spatiotemporal factors towards diversity, and is discussed below.

### Population structure and evolution of DENV-2 genotypes

The population structure of DENV-2 is observed to be complex and is attributed to the high genetic heterogeneity of DENV-2 genotypes. Population genomic analysis helped to reveal that the American and Asian strains of DENV-2 are genetically distinct, emphasizing the primary separation of DENV-2 based on spatial distribution. This observation is also strengthened by an earlier study based on correspondence analysis of complete genomes of DENV-2 (Lara-Ramírez et al., 2014). The present study was found to be particularly useful to understand the lineage diversity within DENV-2 genotypes. Four genotypes of DENV-2 were found to be heterogeneous. The population of DENV-2 strains belonging to AA, AI, C and S genotypes was observed to subdivide further into distinct lineages. The population of other two genotypes (AM and AII) was found to be homogeneous. The AA genotype comprised of strains from both the American and Asian strains. The American strains of AA genotype formed a total of six distinct lineages whereas the Asian AA strains formed independent lineage. The three genotypes such as S, C and AI were comprised exclusively of Asian strains and each was subdivided further into two lineages.

Several key aspects about the genetic heterogeneity within sylvatic genotype were investigated. The sylvatic genotype is characterized by a spatial genetic structure and found to comprise of two genetically distinct lineages (S1 and S2). These lineages possess distinct host range and geographic localities (Africa and Malaysia). It thus supported earlier findings that the Malaysian and African sylvatic DENV-2 strains are genetically distinct (Vasilakis et al., 2008). Apart from spatial subdivision, the present study highlights a significant association between episodic positive selection on several codons in non-structural genes (NS5, NS4B) and evolution of sylvatic strains in general and that of S2 lineage, in particular. The S2 lineage was comprised of two strains from Malaysia, one isolated from a monkey host in 1970 (GenBank: EF105379) while the other (isolate DKD811) was isolated in 2008 and is known to cause Dengue hemorrhagic fever in humans (GenBank: FJ467493). It was deduced that the disease-causing isolate DKD811 of S2 lineage remained undetected and was resident in non-human primates in this location for 38 years (Cardosa et al., 2009). Association of sylvatic DENV-2 strains with disease phenotype in humans has been reported (Cardosa et al., 2009; Franco et al., 2011). Therefore, the mutations in non-structural genes identified with respect to the S2 lineage could be used to monitor such strains in future. Earlier studies reported the potential role of envelope gene in evolution of sylvatic strains (Twiddy, Woelk & Holmes, 2002; Vasilakis et al., 2007). Thus, the observations reported in our study may help to understand the transition of DENV-2 from sylvatic to human hosts mediated through adaptive evolution of the non-structural genes.

In case of Asian I (AI) genotype, the evolution of strains was found to be driven primarily by time and recombination. A clear subdivision of Asian I strains into older AI-1 and modern AI-2 was observed. The Thailand strains isolated during 2001 appear to play an important role in the spread of DENV-2 to modern AI-2 countries such as Cambodia and Vietnam. Several strains of modern AI-2 lineage (from Cambodia and Vietnam) were identified as recombinants (Table 1), having their major parent from Thailand (isolated in 2001) and thus substantiating the role of Thailand strains in spread of DENV-2 in nearby regions. Such conjecture is also supported by an earlier study (Hang et al., 2010). Thailand is observed to act as an epicenter to cause dengue epidemic cycles in the surrounding areas (Cummings et al., 2004; Van Panhuis et al., 2015).

The Cosmopolitan (C) genotype was found to have a hierarchical and spatiotemporal genetic structure wherein all the members were observed to undergo time-dependent subdivision into two lineages such as older C1 and modern C2. The modern C2 lineage is further subdivided into three sub-lineages based on spatial distribution of strains. Such hierarchical genetic structure is attributed to interplay among recombination, episodic positive selection on non-structural genes (NS2B and NS5) and spatiotemporal distribution of cosmopolitan strains. The analysis using the population genetics approach also helped to reveal that the Indonesian strains of modern C2 lineage (GenBank: GQ398263, GQ398264) are admixed having membership for both C1 and C2 subpopulations, thereby emphasizing the role of Indonesia in emergence of DENV-2 in other countries.

The Asian/American (AA) genotype comprised of strains of both Asian and American regions. Interestingly, the AA strains isolated from American region were found to be diversified into a total of six distinct lineages (AA2–AA7) and thus are genetically more heterogeneous as compared to the Asian strains of AA genotype (AA1 lineage). Episodic positive selection on codons of non-structural genes (NS5: codon-558 and NS4B: codon-156) were observed to be operational on AA1 lineage and distinguish AA1 from all the American AA lineages.

These results also indicate that America acts as the diversity hotspot for the DENV-2 serotype. The genetic diversity in Americas is observed to be influenced by spatio-temporal distribution of strains, recombination and episodic positive selection on envelope gene. All the AA strains primarily subdivide based on their geographic location such as Asia, Central America (CAM), South America (SAM) and North America (NAM). NAM lineage (AA2) was formed by the modern strains from USA (2000–2008) whereas older SAM lineage namely AA3 comprised of strains isolated during 1990s from USA as well as SAM countries (Puerto Rico, Venezuela, Brazil). It clearly indicates the transmission of DENV-2 strains from SAM to NAM. Apart from the role of spatio-temporal distribution, the episodic positive selection on two codons on envelope gene (codon-91 and -341) was also found to correlate with the evolution of modern AA lineages in South (AA4 and AA5) and Central America (AA6, AA7). Interestingly, the strains of Asian (AA1) and older SAM (AA3) lineages were observed to have admixture for both the Asian and American subpopulations of AA. This clearly implies that the transmission of DENV-2 strains to other American regions has occurred via countries with the predominance of AA3 isolates. The strains belonging to the AA3 lineage are found to be isolated from the Caribbean region (such as Puerto Rico and Jamaica) and Brazil. This observation was supported by the earlier studies wherein Caribbean islands have been reported to be the main source of DENV-2 viruses in Brazil and northeastern Brazil appears to be an important route of introduction and dissemination of this virus in SAM region (Figueiredo et al., 2014; Mir et al., 2014). Furthermore, gene flow via islands has been proposed to be a cause of spread to other countries in the Americas (Allicock et al., 2012). Thus, the population genetic approach based on allele frequency polymorphisms was found useful to analyze the spread of DENV-2 strains, within American regions.

The diversification of SAM lineages (AA3–AA5) was found to correlate with the temporal distribution of strains. For example, the Brazilian strains isolated during 2000 were clustered into older SAM lineage namely AA3. Further, the Brazilian strains isolated during 2000–2006 were found to cluster within the AA4 lineage whereas the Brazilian strains isolated during 2007–08 clustered within the AA5 lineage. The existence of such temporal structure in case of Brazilian strains is found in accordance with an earlier report (Drumond et al., 2013).

Two lineages of DENV-2 strains from Central America (AA6 and AA7) were observed to contain Nicaragua strains isolated during the same time period i.e., 2005–2009. Such an existence of two co-circulating lineages in Nicaragua is supported by an earlier analysis of DENV-2 (Añez, Morales-Betoulle & Rios, 2011).

Thus, the whole genome-based population genomic analyses helped to reveal the presence of 15 spatio-temporal lineages and examine the role of adaptive evolution and intra-serotype recombination in shaping this structure. It should be noted that genome-based trees were also compared with tree obtained using envelope-gene sequences for the 990 entries. Envelope gene is a commonly used phylogenetic marker for genotyping of DENV-2 and a total of six genotypes of DENV-2 have been documented (Chen & Vasilakis, 2011; Twiddy et al., 2002). Our study aims to examine the diversifying lineages, if any, within six known genotypes. Our studies revealed that DENV-2 genotypes undergo further diversification and there are fifteen distinct lineages. It is noted that the four genotypes are genetically heterogeneous (Asian/American: 7 lineages; Asian I: 2 lineages; Cosmopolitan: 2 lineages and sylvatic: 2 lineages) whereas two genotypes (American and Asian II) are genetically homogeneous and do not show any lineage diversity.

Though, the genome as well as envelope gene-based trees supported the presence of 15 lineages (as shown in Fig. 1), the relative order of clustering was found to vary. The genome-based phylogenetic trees generated using all the three methods (NJ, ML and MP) provided the same relative order of clustering of dengue genotypes such as S-AM-C-AI-AII-AA and indicate that the American genotype was earlier to diverge. On the other hand, the envelope gene-based tree (File S11: Fig. S3) generated using all three methods supported the S-AI/AII-AM-C-AA order. The discrepancy in the relative order of genotypes has been reported earlier based on phylogeny of envelope gene, where either American (AM) or Asian (AI/AII) genotype was found to be the first diversifying urban DENV-2 genotype (Chen & Vasilakis, 2011; Twiddy et al., 2002). Therefore, the comprehensive genome-based phylogenetic analysis helped to resolve the early evolutionary history of DENV-2 population where American genotype was found to diverge earlier than that of other urban genotypes of DENV-2.

### Genetic diversity in DENV-2: implications in antigenicity

Significant evidence of episodic positive selection on all the structural and non- structural genes (except NS4A) is observed, though pervasive positive selection is rare in DENV-2. It indicates that in addition to recombination and spatio-temporal distribution, adaptive evolution also contributes to the diversification of DENV-2 strains or lineages, mainly in S, C and AA genotypes. Episodic positive selection is observed on codons encoding for amino residues known to be part of B-cell or T-cell epitopes. For example, episodic positive selection on two codons in E gene (codon-91 and 340) was found to be stronger on the branches leading to modern lineages (AA4–AA7) of AA genotype in South and Central America. The amino acid residues encoded by these two codons are part of the experimentally known epitopes (Roehrig et al., 1994). It suggests that emergence of strains in Americas is correlated with the antigenicity of structural proteins. Emergence of strains of modern Asian lineages such as C2 is observed to be associated with adaptive evolution operational on codons of non-structural gene (NS2B) which is involved in T-cell immunity.

It should be noted that all but one gene of DENV-2, were found to comprised of codons under significant episodic positive selection. However, in case of DENV-4, a recent population genomics study revealed that the episodic positive selection is confined only to two non-structural genes and the envelope gene (Waman et al., 2016). It thus indicates that in case of DENV-2, antigenic pressure is ascribed to B-cell as well as T-cell mediated immune response as compared to DENV-4 in which antigenic pressure is ascribed T-cell response. Thus, the distinct evolutionary pressures are observed to be operational on distinct Dengue serotypes such as DENV-2 and DENV-4. This provides an explanation for high antigenic diversity of DENV-2 strains which enables adaptation to human host more rapidly, as compared to other DENV serotypes.

## Conclusions

DENV-2 population is comprised of fifteen genetically distinct lineages. The complete genome-based analysis revealed the heterogeneous nature of four genotypes of DENV-2 (Asian/American, Asian I, cosmopolitan and sylvatic). The present study reports the role of episodic positive selection in causing genetic variability in DENV-2 strains and in the emergence of modern lineages of DENV-2. The genotype diversity of DENV-2 strains in American or Asian regions is shaped by the confluence of spatiotemporal distribution, recombination and adaptive evolution. The population genetics approach helped to resolve the uncertainty in classification of recombinant strains.

##  Supplemental Information

10.7717/peerj.2326/supp-1 File S1 The dataset of complete genome sequences from 990 strains of *Dengue virus* serotype 2 used in this study The table lists the assigned serial number, GenBank accession number, strain name, genotype, country, collection date and the subpopulation/lineage. Genotypes are designated as follows: S: Sylvatic, C: Cosmopolitan, AI: Asian I, AII: Asian II, AM: American, AA: Asian/American genotype. In case of non-availability of data, the country or Collection date fields of corresponding entries are given as ‘NA’.

10.7717/peerj.2326/supp-2 File S2 The detailed protocol for the inference of genetic structure of DENV-2 population (using population genetics approach)

10.7717/peerj.2326/supp-3 File S3 The multiple sequence alignment used for phylogenetic analysis The multiple sequence alignment is generated using all the 990 DENV-2 strains and an out-group comprising of complete genome sequences of *Japanese Encephalitis virus* (JEV) [GenBank: NC_001437.1 ], *West Nile virus* (WNV) [GenBank: NC_001563.2 ] and *Murray Valley encephalitis virus* (MVEV) [GenBank: NC_000943.1 ].

10.7717/peerj.2326/supp-4 File S4 The plot of *K* vs & *DeltaK*: determination of optimum number of clusters (*K*_*opt*_) in DENV-2 population ‘*K*’ represents the number of clusters. ‘Δ*K*’ is the rate of change of posterior probability of the data given *K*. The plot is derived to determine optimum number of clusters (*K*_*opt*_) for DENV-2 population (comprising of 990 strains from all the six genotypes). The first major peak of Δ*K* is obtained at *K* = 2 followed by a minor peak at *K* = 15, which clearly indicates the existence of a total of 15 genetically distinct subpopulations within DENV-2 serotype.

10.7717/peerj.2326/supp-5 File S5 Phylogenetic tree of DENV-2 strains obtained using Maximum-Likelihood (ML) method in MEGA Complete genomes of 990 strains of DENV-2 with 1000 bootstrap replicates were used to reconstruct phylogenetic tree using ML method. The fifteen lineages, which are also obtained using STRUCTURE program, are depicted in the tree using color codes as indicated. The (%) bootstrap value associated with each lineage is indicated. There are two lineages (S1 and S2) of Sylvatic genotype, two lineages (C1 and C2) of the cosmopolitan genotype, two lineages (AI-1 and AI-2) of Asian I genotype and a total of seven lineages (AA1–AA7) of Asian/American genotype. The American (AM) and Asian II (AII) genotypes formed independent clusters. AA4* indicates the clade of admixed strains that were found to belong to the AA4 lineage by the STRUCTURE program.

10.7717/peerj.2326/supp-6 File S6 Phylogenetic tree of DENV-2 strains obtained using Maximum-Parsimony (MP) method in MEGA Complete genomes of 990 strains of DENV-2 with 1000 bootstrap replicates were used to reconstruct phylogenetic tree using MP method. The fifteen lineages, which are also obtained using STRUCTURE program, are depicted in the tree using color codes as indicated. The (%) bootstrap value associated with each lineage is indicated. There are two lineages (S1 and S2) of Sylvatic genotype, two lineages (C1 and C2) of the cosmopolitan genotype, two lineages (AI-1 and AI-2) of Asian I genotype and a total of seven lineages (AA1–AA7) of Asian/American genotype. The American (AM) and Asian II (AII) genotypes formed independent clusters. AA4* indicates the clade of admixed strains that were found to belong to the AA4 lineage by the STRUCTURE program.

10.7717/peerj.2326/supp-7 File S7 The plot of *K* vs Δ*K*: determination of optimum number of clusters in sylvatic (S) genotype of DENV-2 ‘*K*’ represents the number of clusters. ‘Δ*K*’ is the rate of change of posterior probability of the data given *K*. The plot is derived to determine optimum number of clusters in sylvatic genotype (comprise of 16 strains) of DENV-2. The major peak of Δ*K* is obtained at *K* = 2, indicates presence of two lineages in sylvatic genotype.

10.7717/peerj.2326/supp-8 File S8 The plot of *K* vs Δ*K*: determination of optimum number of clusters in Asian-I (AI) genotype of DENV-2 ‘*K*’ represents the number of clusters. ‘Δ*K*’ is the rate of change of posterior probability of the data given *K*. The plot is derived to determine optimum number of clusters in Asian I genotype (comprise of 273 strains) of DENV-2. The peak of Δ*K* is obtained at *K* = 2, clearly indicates presence of two lineages in Asian I genotype.

10.7717/peerj.2326/supp-9 File S9 The plot of *K* vs Δ*K*: determination of optimum number of clusters in Cosmopolitan (C) genotype of DENV-2 ‘*K*’ represents the number of clusters. ‘Δ*K*’ is the rate of change of posterior probability of the data given *K*. The plot is derived to determine optimum number of clusters in Cosmopolitan (C) genotype (comprise of 84 strains) of DENV-2. The peak of Δ*K* is obtained at *K* = 4, clearly indicates presence of four sub-clusters in cosmopolitan genotype.

10.7717/peerj.2326/supp-10 File S10 The plot of *K* vs Δ*K*: determination of optimum number of clusters in American-Asian (AA) genotype of DENV-2 ‘*K*’ represents the number of clusters. ‘Δ*K*’ is the rate of change of posterior probability of the data given *K*. The plot is derived to determine optimum number of clusters in American-Asian (AA) genotype (comprise of 552 strains) of DENV-2. The first major peak of Δ*K* is obtained at *K* = 4, followed by a second peak at *K* = 7, indicating presence of substructure and seven distinct lineages in American-Asian genotype.

10.7717/peerj.2326/supp-11 File S11 Envelope gene-based tree of DENV-2 obtained using Neighbor-joining method Envelope gene sequences of 990 strains of DENV-2 with 1,000 bootstrap replicates were used to reconstruct phylogenetic tree using NJ method. The fifteen lineages, which are also obtained using STRUCTURE program, are depicted in the tree using color codes as indicated. There are two lineages (S1 and S2) of Sylvatic genotype, two lineages (C1 and C2) of the cosmopolitan genotype, two lineages (AI-1 and AI-2) of Asian I genotype and a total of seven lineages (AA1–AA7) of Asian/American genotype. The American (AM) and Asian II (AII) genotypes formed independent clusters. AA4* indicates the clade of admixed strains that were found to belong to the AA4 lineage by the STRUCTURE program.

## List of abbreviations

DENV-2: Dengue virus serotype 2
AI: Asian I
AII: Asian II
AM: American
AA: Asian/American
C: Cosmopolitan
S: Sylvatic
MSA: Multiple sequence alignment
AMOVA: Analysis of Molecular Variance
PI: Parsimony-informative
NJ: Neighbor-joining
ML: Maximum likelihood
MP: Maximum parsimony

## Additional Information and Declarations

### Data Availability

The following information was supplied regarding data availability: The raw data has been supplied as a Supplementary File.
