# Supplementary material for: Analysis of genotype diversity and evolution of Dengue virus serotype 2 using complete genomes
Source: PeerJ. 2016 Aug 24;4:e2326. doi: 10.7717/peerj.2326 (PMC5012332; doi:10.7717/peerj.2326)
Supplement: File S2 [file peerj-04-2326-s002.docx]

## Supplemental file 2: The detailed protocol for the inference of genetic structure of DENV-2 population (using Population genetics approach)

The steps involved in the study of genetic structure of *DENV-2* population using population genetics approach include multiple sequence alignment (MSA) of *DENV-2* genomes, extraction of parsimony-informative (PI) sites from MSA, analysis of linkage equilibrium, inference of genetic structure using a model-based population genetics approach and validation of genetic structure using Analysis of Molecular Variance (AMOVA). The details of all these steps are given below.

### Multiple sequence alignment (MSA)

The MSA of 990 *DENV-2* complete genomes were carried out using MUSCLE program (v.8.31) (Edgar AC, 2004).

### Extraction of parsimony-informative (PI) sites

The MSA of 990 *DENV-2* complete genomes, was then used to extract the PI sites from MSA using MEGA v6 (Tamura et al., 2013). A PI site is defined as the site that contains at least two types of nucleotide bases and at least two of them occur with a minimum frequency of two (Tamura et al., 2013). The gaps were considered as the 5^th^ nucleotide state and ambiguous characters were treated as ‘missing values’. The PI sites were referred to as ‘loci’. A total of 4470 PI sites were obtained and used as an input for linkage equilibrium analyses using LIAN 3.5 (Haubold & Hudson, 2000) and inference of population structure using STRUCTURE 2.3.4 (Pritchard & Stephens & Donnelly, 2000; Falush & Stephens & Pritchard, 2003) programs.

### Analysis of linkage equilibrium

Inference of DENV-2 population structure using the STRUCTURE program requires that most of the loci are in linkage equilibrium (Pritchard & Stephens & Donnelly, 2000; Falush & Stephens & Pritchard, 2003). Therefore, the null hypothesis of linkage equilibrium within DENV-2 genomes was tested using LIAN v3.5 (Haubold & Hudson, 2000). The program calculates a standardized index of association, *I^S^_A_*, which is a measure of the degree of haplotype-wide linkage derived from a dataset. The program computes an I^S^_A_ value between parsimony-informative (PI) sites across genomes. In case of free recombination, the value of I^S^_A_ is expected to be zero. For the DENV-2 dataset, the low ISA value of viz. 0.06 (p < 10^-4^, 10000 replicates) was obtained and found to be significant, suggesting weak evidence of linkage disequilibrium (LD).

Two additional measures of linkage disequilibrium (LD) such as |D’| and *r^2^* (Devlin & Risch, 1995) were also computed using DnaSP v5 program (Librado & Rozas, 2009) and by providing complete genome alignment as an input. The measure |D’| represents the absolute value of the difference between the observed and the expected haplotype frequency in the absence of LD, which is normalised by the maximum (or minimum) possible value of this difference. The squared value of the difference between the observed and the expected haplotype frequency normalised by the variance of the allele frequency, is represented by *r^2^*. Consequently, the values of |D’| and r2 were obtained and plotted against the nucleotide distance. Consequently, the plots of |D’| and r2 against physical distance between loci were generated. The low correlation coefficients associated with both |D'| (0.0035) and r^2^ (-0.0011) regressions clearly supports evidence of low LD. Thus the use of STRUCTURE program to study population structure of DENV-2 was found to be suitable as the polymorphic loci are weakly linked.

### Inference of genetic structure

Genetic structure of DENV-2 population was analyzed using a model-based Bayesian clustering approach implemented in the STRUCTURE v2.3.4 program (Pritchard & Stephens & Donnelly, 2000; Falush & Stephens & Pritchard, 2003). The program facilitates identification of genetically distinct subpopulations within a dataset, each of which is characterized by a set of allele frequencies at every locus. The program assigns a membership score (ranging from 0 to 1) to every strain. Membership score of 1 is assigned if an individual belongs to one particular subpopulation. An admixed (recombinant) strain is assigned multiple membership scores (summing to 1) to indicate its membership to multiple subpopulations.

The population structure of DENV-2 was analysed using two models, namely, admixture and linkage with correlated allele frequencies between subpopulations. Such models help to account for individuals having mixed ancestry (potential recombinants) and also probabilistically assign admixed individuals to two or more populations. The linkage model is applied as it accounts for potential linkage between loci and thereby avoids underestimation or overestimation of the admixed individuals (Pritchard & Stephens & Donnelly, 2000; Falush & Stephens & Pritchard, 2003). The admixture model was built using 20,000 burn-in and 40,000 Markov Chain Monte Carlo (MCMC) run lengths. Default values were used for other parameters such as Dirichlet parameter for degree of admixture (*α*) and allele frequency parameter (*λ*). The optimum number of clusters is represented by *K_opt_*. To determine the *K_opt_*, ten independent simulation runs were carried out for each value of *K* ranging from 1 to 19. Ten independent runs for each K were used to verify consistency of parameter estimates across runs and reproducibility of the clusters. These ten replicates helped to obtain the values of posterior probability of data for a given value of *K* and associated standard deviation, which were used to infer *∆K* as suggested in (Evanno & Regnaut & Goudet , 2005). Finally, the value of *K_opt_* is determined by referring to the highest peak in the plot of *K* versus *∆K* (Additional file 1). In order to validate the *K_opt_* obtained suing burn-in of 20000 and burn-length of 40000, an attempt was also made to simulate the data using various combinations of burn-ins and burn-lengths such as, 50000-50000, 70000-70000, and 100000-100000 (Gilks & Richardson & Spiegelhalter, 1996). The linkage model was built using 20,000 burn-in, 40,000 MCMC run lengths and 50,000 admixture burn-in length.

### Validation of genetic structure hypothesis

The population structure in *DENV-2* obtained by STRUCTURE 2.3.4 program was validated using *F*_ST_ values (Fixation indices) obtained by applying AMOVA test implemented in ARLEQUIN 3.11 software (Excoffier & Laval & Schneider, 2005).

**References in Additional file 2:**

- Devlin B, Risch N. A comparison of linkage disequilibrium measures for fine-scale mapping. Genomics. 1995; 29:311-322.
- Edgar RC. MUSCLE: multiple sequence alignment with high accuracy and high throughput. Nucleic Acids Res. 2004;32:1792-1797.
- Evanno G, Regnaut S, Goudet J. Detecting the number of clusters of individuals using the software STRUCTURE: a simulation study. Mol Ecol. 2005; 14:2611-2620.
- Excoffier L, Laval G, Schneider S. Arlequin (version 3.0): an integrated software package for population genetics data analysis. Evol Bioinform Online. 2005; 1:47-50.
- Falush D, Stephens M, Pritchard JK. Inference of population structure using multilocus genotype data: linked loci and correlated allele frequencies. Genetics 2003; 164:1567-1587.
- Gilks W, Richardson S, Spiegelhalter D. Markov Chain Monte Carlo in Practice. New York: Chapman Hall/CRC, 486; 1996.
- Haubold B, Hudson RR. LIAN 3.0: detecting linkage disequilibrium in multilocus data. Bioinformatics 2000; 16:847-849.
- Librado P, Rozas J. DnaSP v5: a software for comprehensive analysis of DNA polymorphism data. Bioinformatics. 2009; 25:1451-1452.
- Pritchard JK, Stephens M, Donnelly P. Inference of population structure using multilocus genotype data. Genetics 2000; 155:945-959.
- Tamura K, Stecher G, Peterson D, Filipski A, Kumar S. MEGA6: molecular evolutionary genetics analysis version 6.0. Mol Biol Evol. 2013; 30:2725-2729.
