# Supplementary material for: Analysis of genotype diversity and evolution of Dengue virus serotype 2 using complete genomes
Source: PeerJ. 2016 Aug 24;4:e2326. doi: 10.7717/peerj.2326 (PMC5012332; doi:10.7717/peerj.2326)

**Supplemental file 4: The plot of *K* vs *ΔK*: determination of optimum number of clusters (K*opt*) in DENV-2 population. ‘**K’ represents the number of clusters. *‘ΔK’* is the rate of change of posterior probability of the data given *K*. The plot is derived to determine optimum number of clusters **(**K_opt_) for DENV-2 population (comprising of 990 strains from all the six genotypes). The first major peak of ΔK is obtained at K=2 followed by a minor peak at K=15, which clearly indicates the existence of a total of 15 genetically distinct subpopulations within DENV-2 serotype.


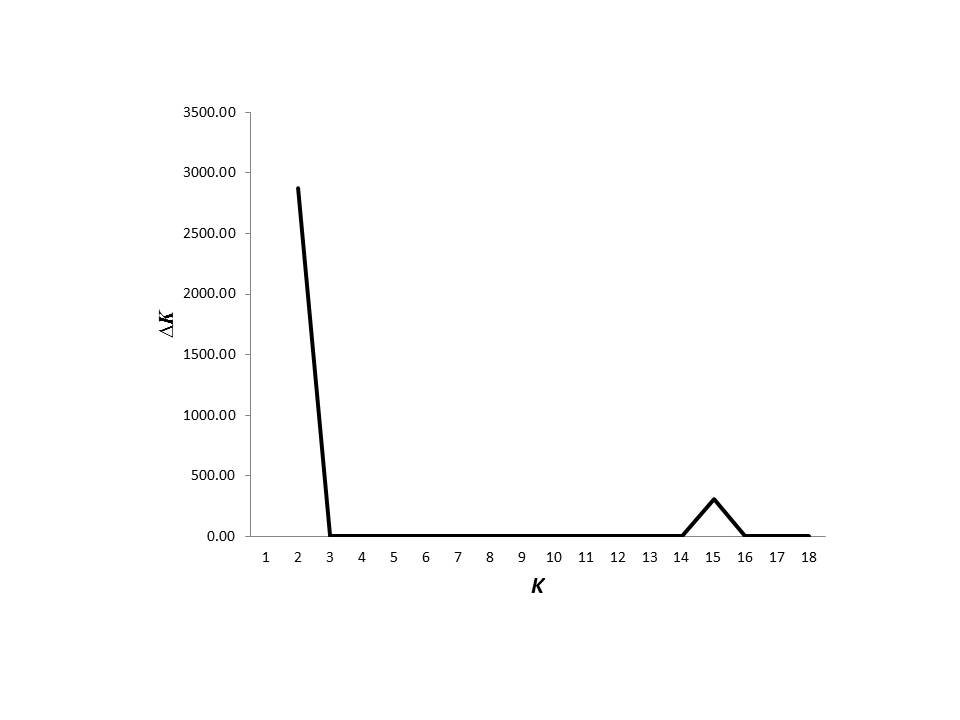

Supplement: File S4 — ‘K’ represents the number of clusters. ‘ΔK’ is the rate of change of posterior probability of the data given K. The plot is derived to determine optimum number of clusters (Kopt) for DENV-2 population (comprising of 990 strains from all the six genotypes). The first major peak of ΔK is obtained at K = 2 followed by a minor peak at K = 15, which clearly indicates the existence of a total of 15 genetically distinct subpopulations within DENV-2 serotype. [file peerj-04-2326-s004.docx]
