# Supplementary figures and images for: Analysis of genotype diversity and evolution of Dengue virus serotype 2 using complete genomes
Source: PeerJ. 2016 Aug 24;4:e2326. doi: 10.7717/peerj.2326 (PMC5012332; doi:10.7717/peerj.2326)

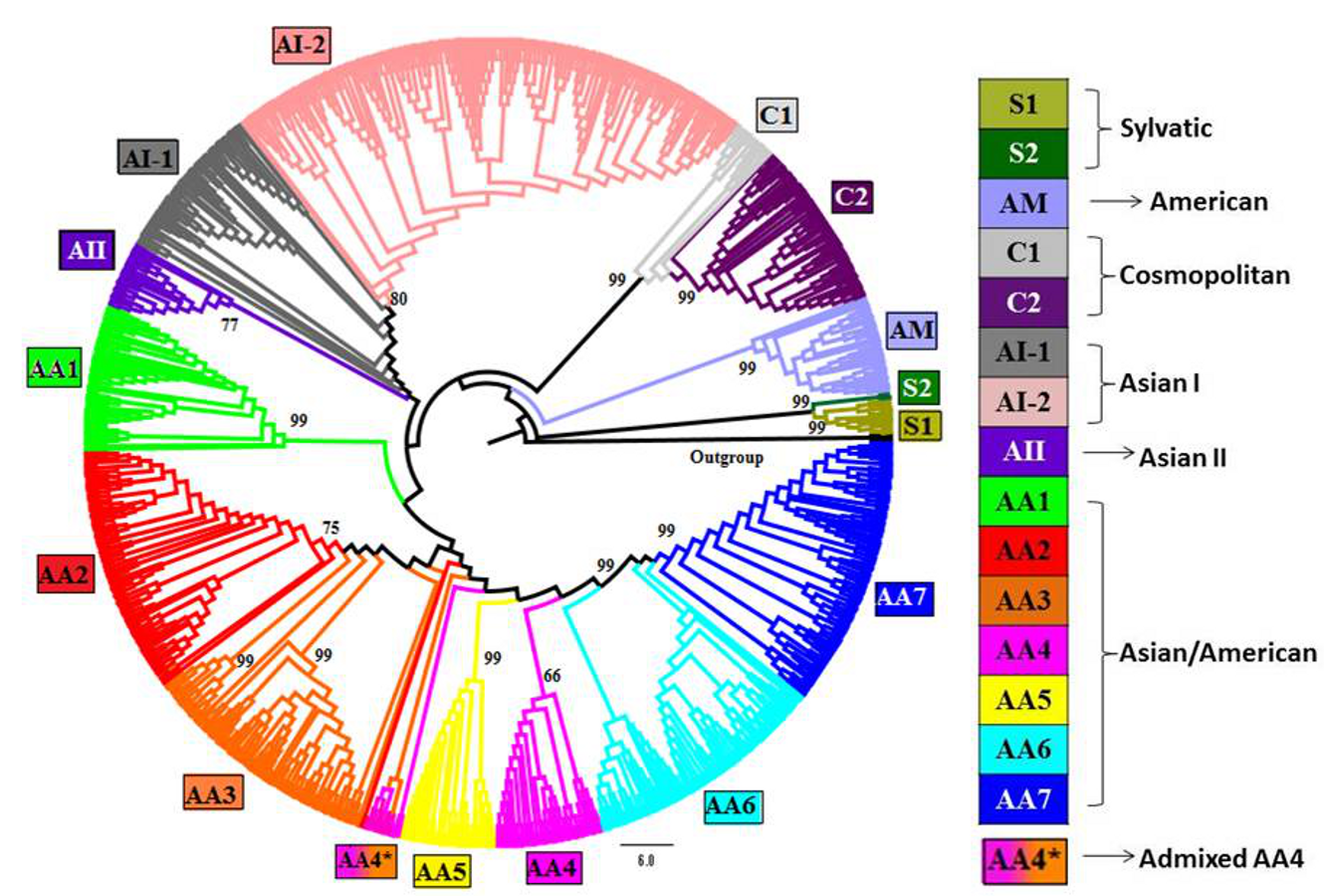

Supplement: File S5 — Complete genomes of 990 strains of DENV-2 with 1000 bootstrap replicates were used to reconstruct phylogenetic tree using ML method. The fifteen lineages, which are also obtained using STRUCTURE program, are depicted in the tree using color codes as indicated. The (%) bootstrap value associated with each lineage is indicated. There are two lineages (S1 and S2) of Sylvatic genotype, two lineages (C1 and C2) of the cosmopolitan genotype, two lineages (AI-1 and AI-2) of Asian I genotype and a total of seven lineages (AA1–AA7) of Asian/American genotype. The American (AM) and Asian II (AII) genotypes formed independent clusters. AA4* indicates the clade of admixed strains that were found to belong to the AA4 lineage by the STRUCTURE program. [file peerj-04-2326-s005.png]

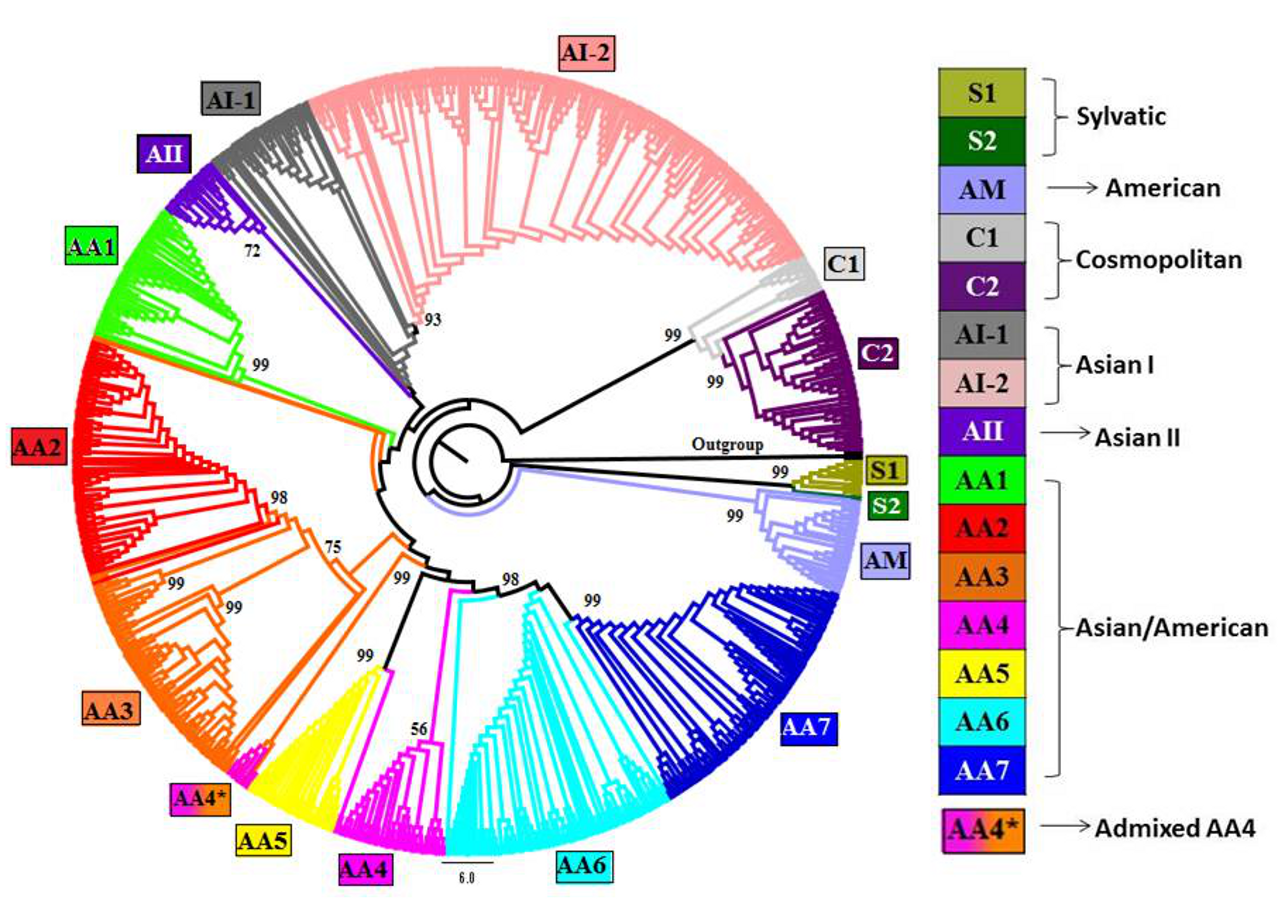

Supplement: File S6 — Complete genomes of 990 strains of DENV-2 with 1000 bootstrap replicates were used to reconstruct phylogenetic tree using MP method. The fifteen lineages, which are also obtained using STRUCTURE program, are depicted in the tree using color codes as indicated. The (%) bootstrap value associated with each lineage is indicated. There are two lineages (S1 and S2) of Sylvatic genotype, two lineages (C1 and C2) of the cosmopolitan genotype, two lineages (AI-1 and AI-2) of Asian I genotype and a total of seven lineages (AA1–AA7) of Asian/American genotype. The American (AM) and Asian II (AII) genotypes formed independent clusters. AA4* indicates the clade of admixed strains that were found to belong to the AA4 lineage by the STRUCTURE program. [file peerj-04-2326-s006.png]

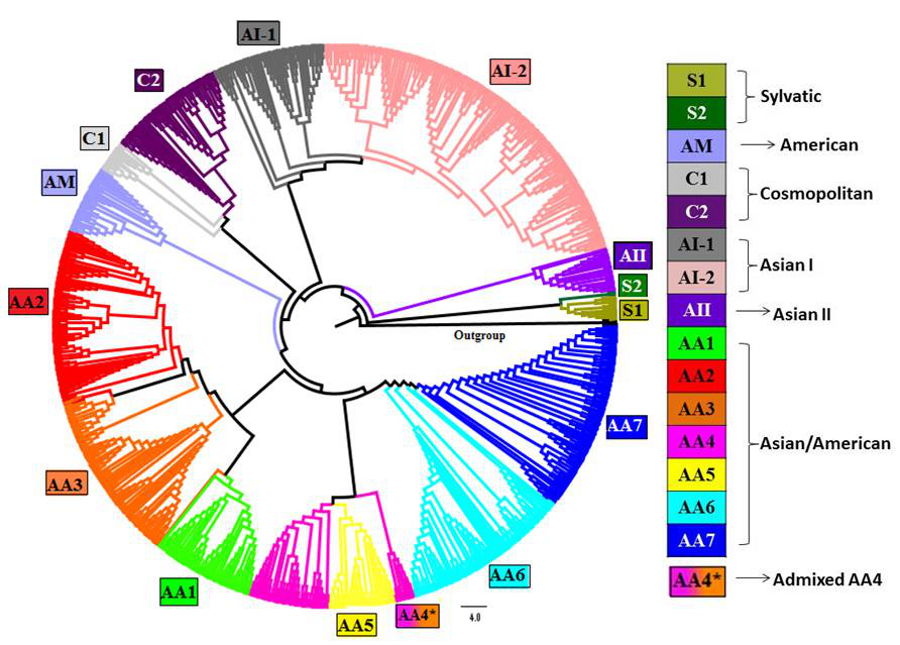

Supplement: File S11 — Envelope gene sequences of 990 strains of DENV-2 with 1,000 bootstrap replicates were used to reconstruct phylogenetic tree using NJ method. The fifteen lineages, which are also obtained using STRUCTURE program, are depicted in the tree using color codes as indicated. There are two lineages (S1 and S2) of Sylvatic genotype, two lineages (C1 and C2) of the cosmopolitan genotype, two lineages (AI-1 and AI-2) of Asian I genotype and a total of seven lineages (AA1–AA7) of Asian/American genotype. The American (AM) and Asian II (AII) genotypes formed independent clusters. AA4* indicates the clade of admixed strains that were found to belong to the AA4 lineage by the STRUCTURE program. [file peerj-04-2326-s011.png]
