# Supplementary material for: Analysis of genotype diversity and evolution of Dengue virus serotype 2 using complete genomes
Source: PeerJ. 2016 Aug 24;4:e2326. doi: 10.7717/peerj.2326 (PMC5012332; doi:10.7717/peerj.2326)

**Supplemental file 8: The plot of *K* vs *ΔK*: determination of optimum number of clusters in Asian-I (AI) genotype of DENV-2.** K’ represents the number of clusters. *‘ΔK’* is the rate of change of posterior probability of the data given *K*. The plot is derived to determine optimum number of clusters in Asian-I genotype (comprise of 273 strains) of DENV-2. The peak of ΔK is obtained at K=2, clearly indicates presence of two lineages in Asian-I genotype.


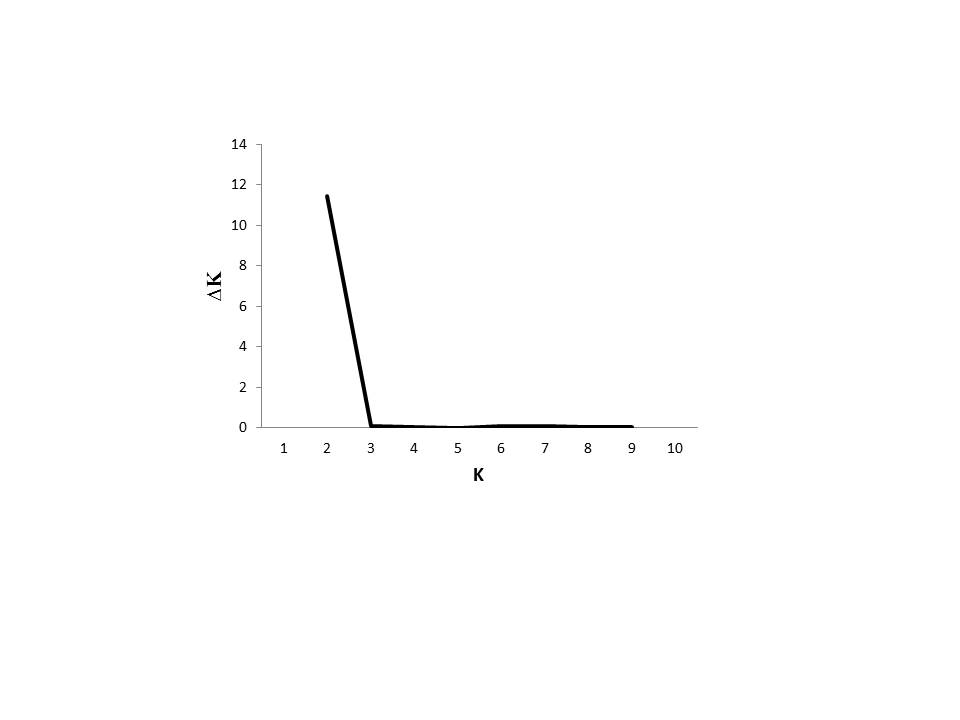

Supplement: File S8 — ‘K’ represents the number of clusters. ‘ΔK’ is the rate of change of posterior probability of the data given K. The plot is derived to determine optimum number of clusters in Asian I genotype (comprise of 273 strains) of DENV-2. The peak of ΔK is obtained at K = 2, clearly indicates presence of two lineages in Asian I genotype. [file peerj-04-2326-s008.docx]
