# Supplementary material for: Analysis of genotype diversity and evolution of Dengue virus serotype 2 using complete genomes
Source: PeerJ. 2016 Aug 24;4:e2326. doi: 10.7717/peerj.2326 (PMC5012332; doi:10.7717/peerj.2326)

**Supplemental file 9: The plot of *K* vs *ΔK*: determination of optimum number of clusters in Cosmopolitan (C) genotype of DENV-2. . ‘**K’ represents the number of clusters. *‘ΔK’* is the rate of change of posterior probability of the data given *K*. The plot is derived to determine optimum number of clusters in Cosmopolitan (C) genotype (comprise of 84 strains) of DENV-2. The peak of ΔK is obtained at K=4, clearly indicates presence of four sub-clusters in cosmopolitan genotype.


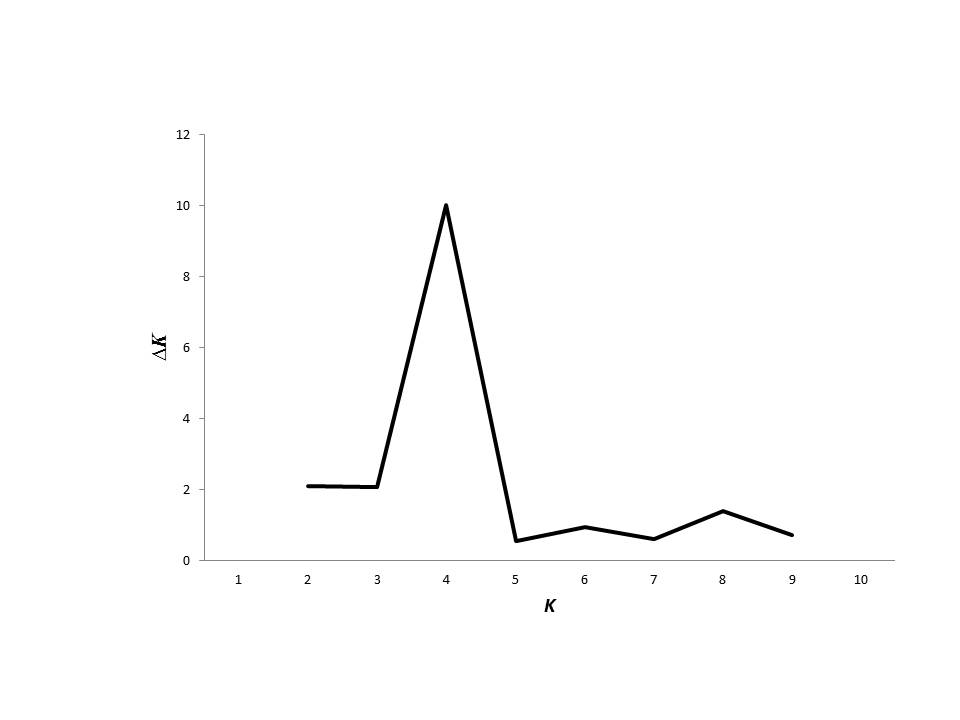

Supplement: File S9 — ‘K’ represents the number of clusters. ‘ΔK’ is the rate of change of posterior probability of the data given K. The plot is derived to determine optimum number of clusters in Cosmopolitan (C) genotype (comprise of 84 strains) of DENV-2. The peak of ΔK is obtained at K = 4, clearly indicates presence of four sub-clusters in cosmopolitan genotype. [file peerj-04-2326-s009.docx]
