# Supplementary material for: Analysis of genotype diversity and evolution of Dengue virus serotype 2 using complete genomes
Source: PeerJ. 2016 Aug 24;4:e2326. doi: 10.7717/peerj.2326 (PMC5012332; doi:10.7717/peerj.2326)

**Supplemental file 10: The plot of *K* vs *ΔK*: determination of optimum number of clusters in sylvatic (S) genotype of DENV-2. . ‘**K’ represents the number of clusters. *‘ΔK’* is the rate of change of posterior probability of the data given *K*. The plot is derived to determine optimum number of clusters in sylvatic genotype (comprise of 16 strains) of DENV-2. The major peak of ΔK is obtained at K=2, supports presence of two lineages in sylvatic genotype.


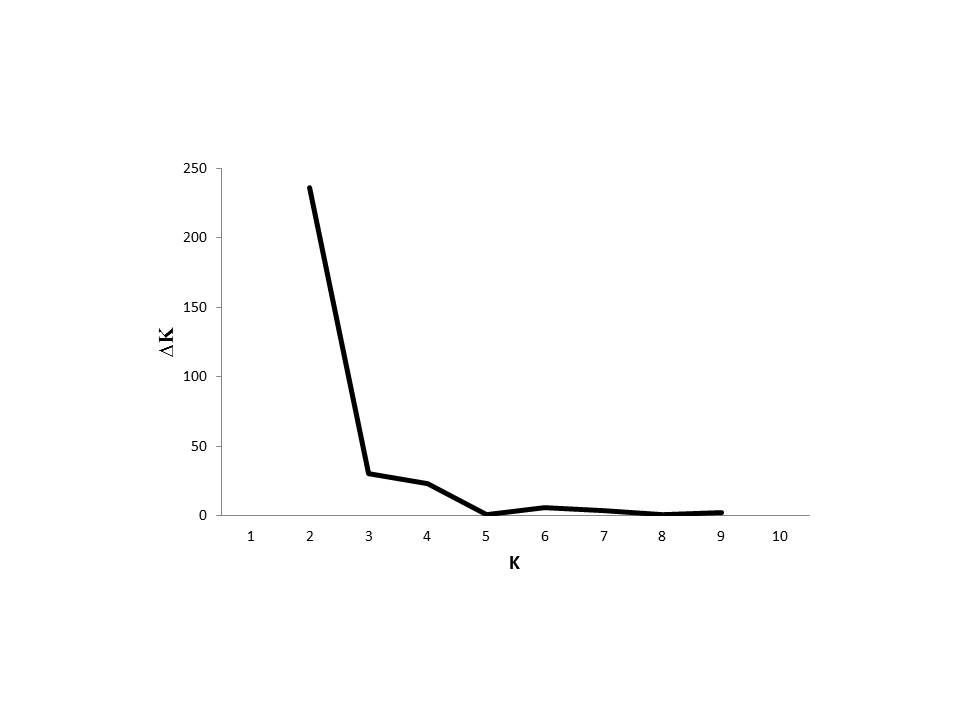

Supplement: File S10 — ‘K’ represents the number of clusters. ‘ΔK’ is the rate of change of posterior probability of the data given K. The plot is derived to determine optimum number of clusters in American-Asian (AA) genotype (comprise of 552 strains) of DENV-2. The first major peak of ΔK is obtained at K = 4, followed by a second peak at K = 7, indicating presence of substructure and seven distinct lineages in American-Asian genotype. [file peerj-04-2326-s010.docx]
